# A Novel Polysaccharide from Walnut Dregs: Structural Features and Immunomodulatory Effects via Activation of MAPK Signaling Pathway

**DOI:** 10.3390/foods15132252

**Published:** 2026-06-23

**Authors:** Wanying Gong, Min Su, Tianyi Dai, Jinlian Chen, Qianqian Yang, Li Zhang, Wenjing Wang, Weitao Zhang, Jun Sheng, Jing Xie, Yang Tian

**Affiliations:** 1 College of Food Science and Technology, Yunnan Agricultural University, Kunming 650201, China; gongwanying1206@163.com (W.G.); 17336694755@163.com (M.S.); dtynongda@163.com (T.D.); 18395180270@163.com (J.C.); yqqhappy@126.com (Q.Y.); zhangl25@126.com (L.Z.); wwjynau@163.com (W.W.); zwt13887797821@163.com (W.Z.); shengjun_ynau@163.com (J.S.); 2 College of Tea (Pu’er), West Yunnan University of Applied Sciences, Pu’er 665000, China; 3 Engineering Research Center of Development and Utilization of Food and Drug Homologous Resources, Ministry of Education, Yunnan Agricultural University, Kunming 650201, China; 4 Yunnan Provincial Key Laboratory of Precision Nutrition and Personalized Food Manufacturing, Yunnan Agricultural University, Kunming 650201, China; 5 Yunnan Provincial Engineering Research Center for Edible and Medicinal Homologous Functional Food, Yunnan Agricultural University, Kunming 650201, China; 6 Pu’er Institute of Pu-erh Tea, Pu’er 665000, China

**Keywords:** walnut dregs, polysaccharide, isolation, purification, structural characterization, immunomodulatory activity

## Abstract

A novel acidic polysaccharide (WDP) was purified from walnut dregs, and its structural characteristics and immunomodulatory function were investigated. WDP had a weight-average molecular weight (Mw) of 351.94 kDa and consisted mainly of rhamnose, arabinose, galactose, glucose, xylose, mannose, galacturonic acid, and glucuronic acid. Methylation and NMR analyses further demonstrated that the backbone of WDP comprised →4)-α-D-GalpA-(1→, →3,6)-β-D-Galp-(1→, →6)-β-D-Galp-(1→, →4)-β-D-Galp-(1→, and →4)-α-D-Glcp-(1→ residues, with branched chains consisting of terminal α-L-Araf-(1→ residues or α-L-Araf-(1→5)-α-L-Araf-(1→ fragments attached to the O-3 position of →3,6)-β-D-Galp-(1→ residues. In vitro assays indicated that WDP modulated immune responses in RAW264.7 cells by enhancing their phagocytosis; increasing NO release and the secretion of IL-1β, IL-6 and TNF-α; and activating the MAPK signaling pathway, suggesting its potential as an immunomodulatory agent. These results provide a scientific foundation for the development of walnut dregs-derived functional foods with immune-enhancing properties.

## 1. Introduction

The immune system represents a sophisticated defensive network, comprising cells, tissues, and organs that collaboratively safeguard the host from infectious agents, thereby maintaining health and preventing disease [1]. The immune system serves a crucial function in modulating immunological responses and sustaining immune homeostasis, which may be disrupted by multiple influences such as malnutrition, stress, an unhealthy lifestyle, and chemotherapy, potentially contributing to the development of diverse disorders [2,3]. Modulating immune responses is considered central to preventing illness [4], and the main immunomodulators currently in use are drugs such as cyclophosphamide, glucocorticosteroids, histamine antagonists, and non-steroidal anti-inflammatory drugs. Nonetheless, these medications come with certain toxic side effects [4,5], and therefore the pursuit of safer and more reliable immunomodulators is essential.

As a natural component of plants, polysaccharides have been widely reported by numerous studies in recent years as a class of natural immunomodulators with great potential. Polysaccharides from plants have beneficial immunomodulatory properties and have fewer side effects than traditional chemical drugs. These properties make them attractive candidates in the field of immunomodulation. Consequently, polysaccharides have been evaluated as new immunomodulatory agents and could potentially replace some chemical drugs [6,7,8]. Polysaccharides exert immunomodulatory effects mainly by activating macrophages, T and B lymphocytes, the reticuloendothelial system, and the complement system, thereby augmenting the functional activity of immune cells and enhancing the release of pro-inflammatory cytokines. Additionally, they can engage immune cells via specific receptor-mediated recognition and trigger downstream signaling cascades, thereby inducing immunomodulatory responses [9,10,11].

Walnuts (*Juglans regia* L.) are highly regarded for their nutritional profiles and are classified as healthy food. Walnut kernels are often eaten as a convenient snack and enjoyed for their distinct flavor and texture. Walnut kernels also serve as an important raw material for walnut oil, which is praised for its health benefits. During walnut oil production, walnut dregs are produced as a by-product. They are rich in nutrition and have the potential to develop into value-added products [12]. However, despite their potential, the usage rate of walnut dregs is low. They are usually discarded or used solely as animal feed, resulting in food resource waste. Research has indicated that walnut dregs are abundant in proteins and also contain polysaccharides. Like polysaccharides from other plant sources, these polysaccharides may exhibit various biological activities [13]. Therefore, walnut dregs can be a cheap source of polysaccharides, and the extraction of these polysaccharides offers considerable commercial value and promising application prospects. Notably, polysaccharides from other walnut-derived materials, such as *Diaphragma juglandis fructus* (the woody septum inside the walnut) and walnut green husk, have demonstrated good immunomodulatory activity [14,15]. These findings suggest that walnut-derived by-products may be promising sources of immunomodulatory polysaccharides. However, thus far, systematic studies on the isolation, structural characterization, and immunomodulatory evaluation of polysaccharides derived from walnut dregs remain limited.

In this paper, walnut dregs polysaccharide (WDP) was isolated from walnut dregs by water extraction followed by ion-exchange and gel-filtration chromatography. The structure of WDP was deduced using a combination of techniques including molecular weight analysis, ultraviolet (UV) spectroscopy, monosaccharide composition analysis, Fourier-transform infrared spectroscopy (FT-IR), methylation analysis, scanning electron microscopy (SEM), and nuclear magnetic resonance (NMR). Immunomodulatory evaluation of WDP was conducted in RAW264.7 cells by measuring cell viability, phagocytosis, nitric oxide (NO), and cytokine levels. Finally, the signaling pathway through which WDP exerted its effects was evaluated.

## 2. Materials and Methods

### 2.1. Materials

Walnut (*Juglans sigillata* Dode) dregs were from Dali Yunshang Purui Agricultural Co., Ltd. (Dali, China). The Sephacryl S-400HR was from GE (Beijing, China). DEAE seplife FF was obtained from Xi’an Lanxiao Science and Technology New Material Co., Ltd. (Xi’an, China). RAW264.7 cells were sourced from the Kunming Institute of Zoology, Chinese Academy of Sciences (Kunming, China); thiazolyl blue (MTT), phosphate buffer salt (PBS) solution, and lipopolysaccharide (LPS) were from Beijing Solebao Technology Co. Ltd. (Beijing, China); Elisa kits were obtained from Jiangsu Enzyme Immunity (Yancheng, China); and Griess Reagent System NO Detection Kit was from Promega (Madison, WI, USA).

### 2.2. Extraction and Purification of WDP

The extraction and purification of WDP were performed according to previous studies with slight modifications [16,17]. Briefly, walnut dregs were initially milled into a powder and sifted with a 60-mesh screen. After that, the powder was mixed with 10 volumes of anhydrous ethanol to remove the contaminants that dissolve in lipids, then centrifuged (6000× *g*, 10 min) and the precipitate was collected. The dried precipitate was extracted twice with distilled water (1:20, *w*/*v*) at 60°C for 4 h. After each extraction, the mixture was centrifuged (6000× *g*, 10 min) and the supernatants were collected, combined, concentrated, and precipitated with four volumes of anhydrous ethanol at 4 °C overnight to obtain the crude polysaccharide precipitate. The crude polysaccharide precipitate was then resuspended in distilled water, treated with papain overnight for enzymatic protein removal, and further deproteinized by the Sevag method. The upper aqueous phase was collected and extracted with petroleum ether to remove residual lipid impurities. After thorough mixing and phase separation, the lower aqueous phase was collected. Subsequently, macroporous resin AB-8 was added to the aqueous fraction, mixed thoroughly, and allowed to adsorb overnight to remove pigments and small hydrophobic impurities. The solution was collected and successively dialysed (3 kDa) against water for 48 h, concentrated, and lyophilized to produce crude WDP.

The crude WDP was fractionated on a DEAE Seplife FF column (26 mm × 400 mm) which was eluted sequentially with distilled water, followed by progressively increasing NaCl concentrations (0.1, 0.2 and 0.3 M) at 4 mL/min. Each 15 mL of eluent was collected and its carbohydrate content was quantified using the phenol–sulfuric acid method [18], with the elution curve subsequently plotted. Fractions with high polysaccharide content were pooled, concentrated, and subsequently dialysed (3 kDa) against water for 48 h to remove salt impurities. After that, the purified polysaccharide solution was introduced into a Sephacryl S-400HR column (26 mm × 1000 mm) [19], which was eluted with distilled water at 1.0 mL/min. Every 12 mL of eluent was collected, the total carbohydrate content was determined, and the gel purification elution curve was plotted. The eluates from the identical elution peak were gathered, evaporated, and lyophilized.

### 2.3. Chemical Composition of WDP

The total carbohydrate content and uronic acid content were measured using the phenol–sulfuric acid method and the carbazole-sulfuric acid method, respectively [20].

### 2.4. Structural Characterization of WDP

Homogeneity and Mw were measured according to the published methods [21,22]. The aqueous solution of the WDP was characterized via its UV–visible absorption spectrum from 200 to 500 nm by a microplate reader (Thermo Fisher Scientific, Waltham, MA, USA). The monosaccharide constituents of the WDP were analyzed by the HPAEC-PAD method [23]. The FT-IR spectroscopy of the WDP was performed using a spectrometer (Nicolet iZ-10, Thermo Nicolet, Madison, WI, USA) [19]. The microscopic architecture of the WDP was examined by SEM (Zeiss Merlin Compact, Carl Zeiss, Jena, Germany). The methylation analysis of the WDP followed the procedures outlined in the reference [24]. Prior to methylation, the WDP samples were independently reduced with NaBH_4_ or NaBD_4_. The systematic NMR analysis of the WDP, including 1D and 2D spectra, was performed using a Bruker AVANCE NEO 500 M spectrometer (Bruker, Rheinstetten, Germany) to obtain complete structural information. Detailed methodological procedures are stated in the Supplementary Material.

### 2.5. Studies on Immunomodulatory Effects

#### 2.5.1. Cell Culture

The RAW264.7 cells were cultured in complete DMEM in a humidified incubator at 37 °C with 5% CO_2_.

#### 2.5.2. Cell Viability Assay

The cells were plated into 96-well plates (2 × 10^4^ cells per well) and incubated for 24 h, after which the cells were treated with the WDP (25–100 μg/mL) or LPS (1 μg/mL) for an additional 24 h. A blank control (culture medium only) was also included in the experimental design. After treatment, the cells were subjected to an MTT assay to determine viability [25].

#### 2.5.3. Cellular Phagocytosis and NO Secretion Assays

The cells were treated with WPD (25–100 μg/mL) or LPS (1 µg/mL) according to procedures detailed in Section 2.5.2. After treatment, their phagocytic capacity was assessed via a neutral red uptake assay [26]. NO release by the cells was determined using the Griess Reagent System Nitric Oxide Detection Kit, and NO levels in cell culture supernatants were determined as per the manufacturer’s instructions.

#### 2.5.4. Cellular Cytokines Secretion Assays

The cells were plated in 6-well plates (4 × 10^5^ cells/well). After attachment, the cells were treated with the WDP (25–100 μg/mL) or LPS (1 µg/mL) for 24 h. The concentrations of TNF-α, IL-6, and IL-1β in the culture supernatants were then quantified using ELISA kits according to the manufacturer’s protocols.

#### 2.5.5. Cellular mRNA Levels of *iNOS* and Cytokines

As described in Section 2.5.4, the RAW264.7 cells were seeded, cultured, and treated with the WDP (25–100 μg/mL) or LPS (1 µg/mL). After treatment, total cellular RNA was extracted, converted into cDNA through reverse transcription, and subsequently subjected to RT-qPCR analysis. All the primer sequences for the target genes were provided in Table S1.

#### 2.5.6. Cellular Expression Levels of MAPK Signaling Pathway-Related Proteins

To investigate the immunomodulatory mechanism of the WDP, the phosphorylation status of key MAPK pathway proteins was examined using Western blot (WB) analysis. Following the procedures outlined in Section 2.5.4, the cells were subjected to seeding, subsequent culturing, and final treatment with the WDP (25–100 μg/mL) or LPS (1 µg/mL). Subsequently, the cells were lysed using ice-cold RIPA lysis buffer that contained 1 mM PMSF in the plates to collect total cellular protein, and the levels of expression for target proteins in these cells, including the total and phosphorylated forms of ERK, JNK, p38 MAPK, along with β-tubulin as a loading control, were examined by Western blot analysis following established procedures outlined in prior studies [27].

### 2.6. Statistical Analysis

All the experiments described in Section 2.5 were performed in triplicate or more and the experimental data were expressed as mean ± standard deviation (x ± SEM). One-way ANOVA was performed with GraphPad Prism 9.0 (GraphPad Software, LLC, Boston, MA, USA); differences were considered statistically significant at *p* < 0.05.

## 3. Results and Discussion

### 3.1. Extraction, Isolation and Purification of WDP

**Figure 1:**
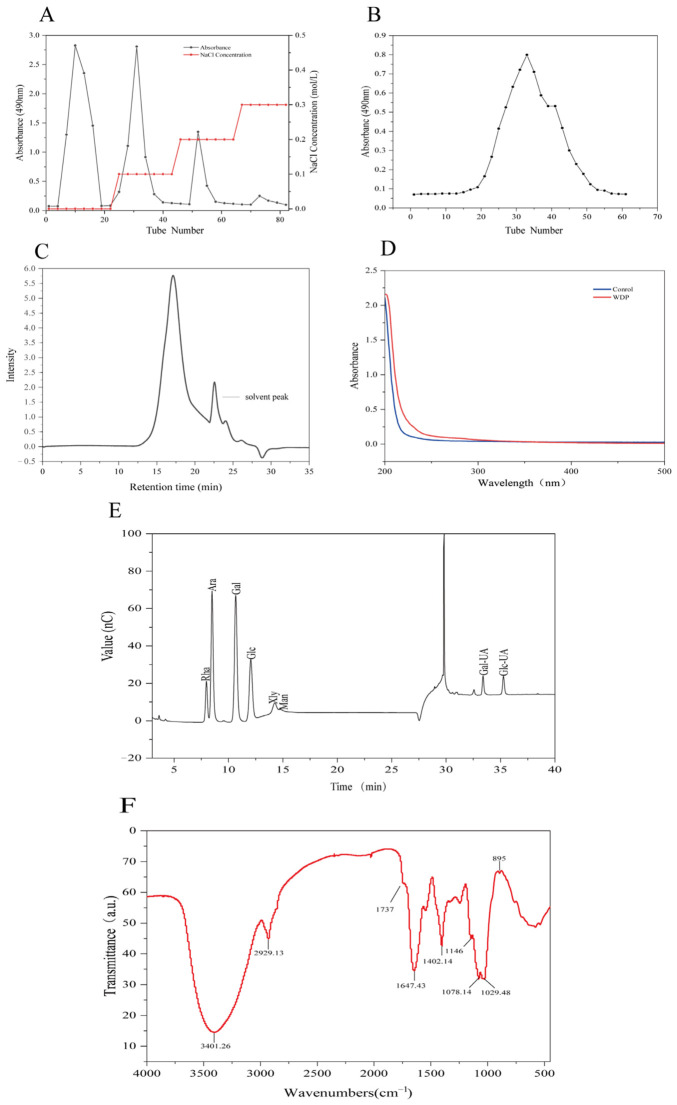
Structural characterization of WDP: (**A**) DEAE seplife FF elution profile of walnut dregs crude polysaccharide. (**B**) Sephacryl S-400HR of walnut dregs crude polysaccharide. Eluted with distilled water. (**C**) The HPSEC of WDP. (**D**) UV spectroscopy of WDP. (**E**) Monosaccharide composition of WDP. (**F**) FT-IR spectrum of WDP.

As presented in Figure 1A, single elution peaks appeared at 0, 0.1, and 0.2 M NaCl eluents, yielding three fractions. The first fraction had the largest peak area. With increasing eluent concentration, the peak area decreased sequentially, and the three fractions were collected, concentrated, dialyzed and lyophilized to determine their contents, and the carbohydrate contents were 71.10%, 67.60% and 53.80%, in that order. The polysaccharide fraction eluted with distilled water had the highest purity and the highest yield of 35.97% (relative to the crude polysaccharide form walnut dregs). Therefore, the distilled water-eluted fraction was further purified on a Sephacryl S-400HR column; the WDP was finally obtained (Figure 1B) with a carbohydrate content of 90.90%, and the content of uronic acid was 21.64%. Uronic acids encompass key carboxylic acid derivatives, including galacturonic acid, glucuronic acid, and mannuronic acid, which are important components of complex polysaccharides [28]. Previous studies have suggested that polysaccharides containing uronic acid may exhibit immunomodulatory activity, and an appropriate uronic acid ratio for immune enhancement was approximately 20–50% [29]. In this study, the uronic acid content of the WDP was 21.64%, indicating that WDP was an acidic polysaccharide and suggesting that uronic acid might be one of the structural features associated with its potential immunomodulatory activity.

### 3.2. Homogeneity and Molecular Weight of WDP

As shown in Figure 1C, the HPSEC chromatogram of the WDP displayed a predominant single peak, suggesting that the WDP was relatively homogeneous, and the Mw of the WDP was 351.935 kDa. Compared with the polysaccharides extracted from walnut green husk and walnut shell, the WDP had a higher molecular weight [15,30,31].

### 3.3. Ultraviolet Spectral Analysis of WDP

The absorption spectra typically exhibit distinct peaks for nucleic acids and proteins at 260 nm and 280 nm, respectively. However, as illustrated in Figure 1D, no such characteristic peaks were observed at these wavelengths, indicating that the WDP solution was free from protein and nucleic acid impurities.

### 3.4. Monosaccharide Composition of WDP

The monosaccharides in the WDP were analyzed by comparing the sample and standard ion chromatograms, and the result is presented in Figure 1E. The WDP was primarily composed of rhamnose, arabinose, galactose, glucose, xylose, mannose, galacturonic acid and glucuronic acid in a molar ratio of 9.41:28.76:25.90:13.87:6.42:2.90:8.18:4.55. Previous studies have reported that walnut green husk polysaccharides are richer in rhamnose and galacturonic acid [30], walnut green husk acidic polysaccharides contain higher proportions of galactose and galacturonic acid [15], and walnut shell polysaccharides are richer in galactose and galacturonic acid [31], while the WDP had a higher proportion of arabinose and galactose. The composition and molar ratio of monosaccharides influence the biological function of polysaccharides. The major monosaccharides in the WDP were arabinose, galactose, and glucose, followed by galacturonic acid and rhamnose, with low proportions of xylose, glucuronic acid, and mannose. Polysaccharides with high levels of arabinose and galactose enhance immunomodulatory activities [32,33]. Rhamnose and mannose stimulate cellular cytokine production and enhance cellular phagocytosis [34]. Therefore, as inferred from the monosaccharide composition, the WDP might have good immunomodulatory activity.

### 3.5. FT-IR Spectroscopy Analysis of WDP

The chemical functional groups present in the WDP were identified employing FT-IR. According to Figure 1F, a broad band appeared near 3401.26 cm^−1^, which corresponded to the O-H stretching vibrations. The spectral signal at 2929.13 cm^−1^ corresponded to C-H stretching vibrations; these two peaks represent the signature peaks for sugars. The simultaneous presence of a weak absorption at 1737 cm^−1^ and a strong absorption at 1647.43 cm^−1^ indicated the existence of both methyl-esterified and ionized forms of –COOH, respectively, which is a characteristic of uronic acid-containing polysaccharides. The spectral signal at 1402 cm^−1^ was related to the symmetrical stretching vibration of the COO^−^ groups, indicating the presence of uronic acid [35,36,37]. The characteristic peak at 1146 cm^−1^ was related to the C-O-C stretching vibration within glycosidic linkages. The spectral signals at 1078.14 cm^−1^ and 1029.43 cm^−1^ were attributed to C-O-H stretching vibrations within pyranose ring configurations [19]. Additionally, the signal at 895 cm^−1^ was indicative of β-glycosidic linkages, which is a typical polysaccharide structural feature with immunostimulatory characteristics [36].

### 3.6. Morphological Examination of WDP by Scanning Electron Microscopy (SEM)

**Figure 2:**
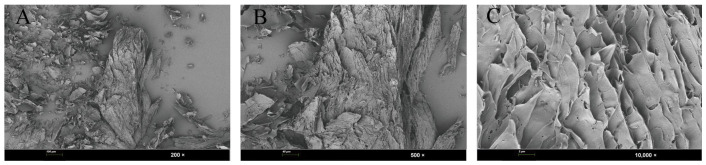
SEM of WDP. States of WDP at 200× (**A**), 500× (**B**) and 10,000× (**C**).

SEM was used to observe the surface morphology of WDP at different magnifications. The image at 200× showed the overall morphology and aggregation state of the WDP, while the image at 500× further revealed its lamellar stacking structure. At 10,000×, the WDP displayed a wrinkled and rough surface with lamellar-like features, distinct undulations, and grooves. Overall, the WDP exhibited an irregular lamellar and stacked morphology with a rough surface (Figure 2). These features may be related to the aggregation and dehydration of polysaccharide chains during ethanol precipitation and lyophilization. The lamellar structure and rough surface may increase the contact area between the WDP and water, thereby facilitating its hydration and dispersion behavior [38].

### 3.7. Methylation Analysis of WDP

Glycosidic linkages in the WDP were characterized using methylation analysis coupled with GC-MS, and the results are included in Table 1. Methylation analysis is an important method for polysaccharide structural characterization, as it provides information on the linkage positions and branching patterns of monosaccharide residues by identifying partially methylated alditol acetates generated after methylation, hydrolysis, reduction, and acetylation.

**Figure 3:**
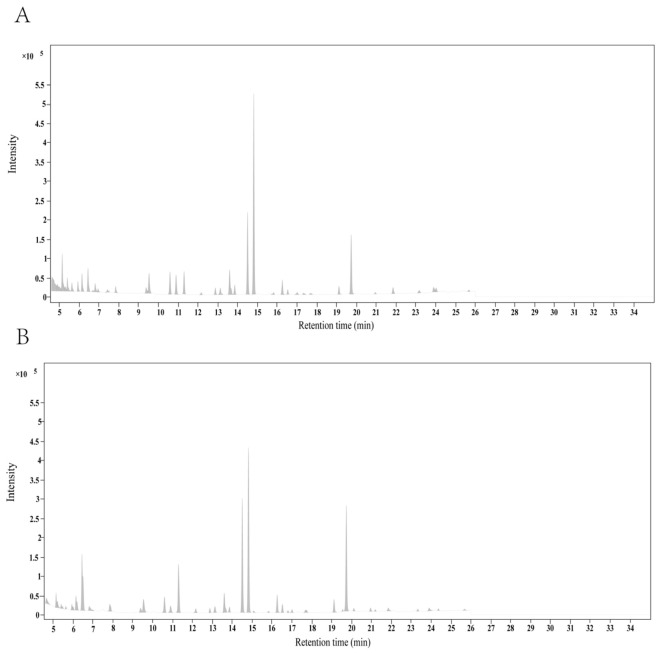
Total ion chromatograms of methylated WDP derivatives: (**A**) D-reduced derivatives, (**B**) H-reduced derivatives.

The glycosidic linkage patterns in the WDP were identified based on the total ion chromatograms (TIC) of the D- and H-reduced methylated derivatives (Figure 3), together with their characteristic MS fragmentation patterns (Figure S1). By comparing the retention times of the peaks together with their diagnostic fragment ions with those in standard reference data, the glycosidic linkages determined in WDP included: t-Rha(p), t-Ara(f), t-Xyl(p), 2-Rha(p), t-Man(p), 5-Ara(f), 3-Gal(p), 4-Gal(p)-UA, 4-Gal(p), 4-Glc(p), 2,4-Xyl(p), 6-Gal(p), 3,4-Glc(p)-UA, 4,6-Glc(p), and 3,6-Gal(p) (Table 1). In the methylation analysis, the WDP contained 27.58% galactose, 27.02% arabinose, 21.9% glucose, 12.3% galacturonic acid, 4.42% rhamnose, 3.09% xylose, 2.88% mannose, and 0.8% glucuronic acid, which was generally consistent with its monosaccharide composition. Further, the most abundant linkages were in the following order: 4-Glc(p) (19.68%), t-Ara(f) (15.32%), 3,6-Gal(p) (14.05%), 4-Gal(p)-UA(12.3%) and 5-Ara(f) (11.7%), which suggested that these linkages represent major structural features of the WDP. The high proportion of 4-Glc(p) indicated that →4)-Glcp-(1→ was an abundant linear linkage type in WDP, while 4-Gal(p)-UA suggested the presence of →4)-GalpA-(1→ residues, which are commonly associated with acidic polysaccharide regions. The relatively high abundance of t-Ara(f) and 5-Ara(f) indicated the presence of terminal arabinofuranose residues and arabinan-related side chains. In addition, the presence of 3,6-Gal(p) suggested that some galactose residues were substituted at both O-3 and O-6, indicating potential branching points. Therefore, methylation analysis indicated that WDP possessed a complex branched structure composed of multiple linkage types, providing important evidence for further structural assignment by NMR analysis.

### 3.8. Nuclear Magnetic Resonance Analysis of WDP

**Figure 4:**
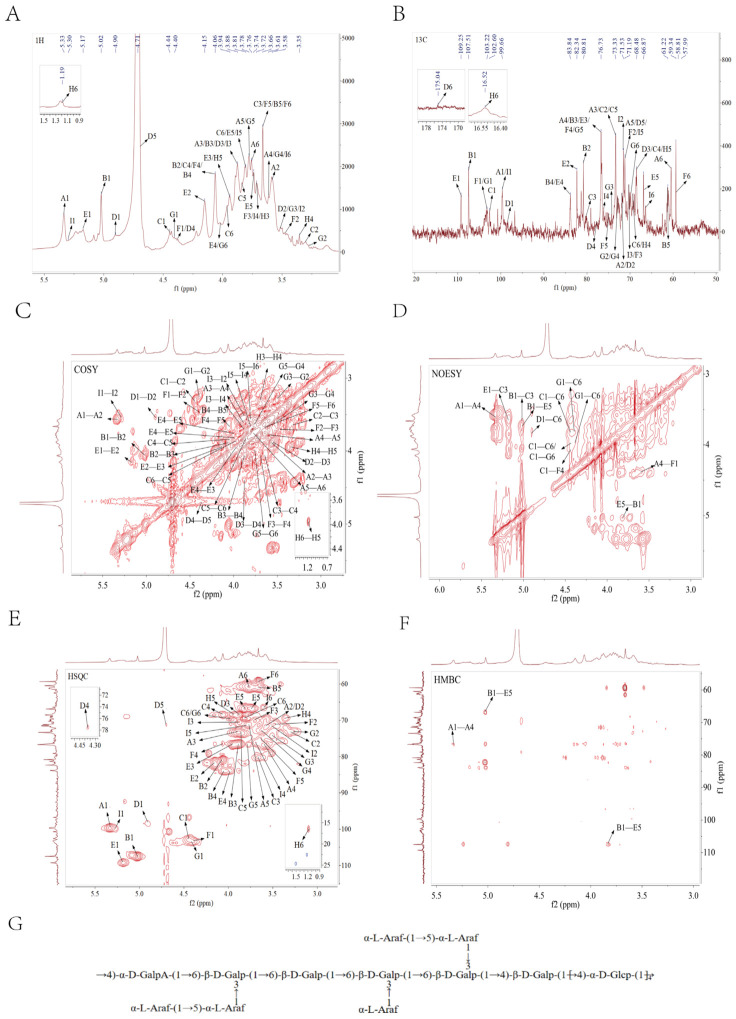
The 1D NMR and 2D NMR spectra of WDP: (**A**) ^1^H-NMR, (**B**) ^13^C-NMR, (**C**) COSY, (**D**) NOESY, (**E**) HSQC, (**F**) HMBC, (**G**) The deduced chemical structure of WDP.

Comprehensive structural elucidation of WDP was achieved through 1D (^1^H, ^13^C; Figure 4A,B) and 2D NMR experiments (COSY, NOESY, HSQC, HMBC; Figure 4C–E); chemical shifts (δ) were recorded in ppm. In the ^1^H NMR spectrum (Figure 4A), signals were predominantly clustered within δ 3.0–5.5. Multiple coupled resonances in the anomeric region (δ 4.3–5.4) indicated various sugar residues, with anomeric proton chemical shifts at δ 4.38, 4.39, 4.44, 4.91, 5.02, 5.17, 5.29, and 5.33. Consistent with established conventions, anomeric protons of β-configuration typically resonate at δ 4.3–4.9, whereas those of α-configuration are observed in the δ 4.9 to 5.8 region [39], suggesting that both α- and β- configurations may coexist in WDP. Non-anomeric proton signals (δ3.1–4.2) exhibit severe overlap in certain regions, necessitating complementary COSY and HSQC spectra for complete H2–H6 assignments. The intense resonance at δ 4.71 is attributed to the solvent peak.

Multiple signals observed in the anomeric carbon region were cross-validated using ^13^C NMR and HSQC anomeric cross-peaks, leading to the assignment of eight anomeric pairs: δ 5.33/99.67, 5.02/107.52, 4.44/102.60, 4.91/98.37, 5.17/109.25, 4.38/103.22, 4.39/103.44, and 5.29/99.28 (δ_H/δ_C). These residues were designated as A, B, C, D, E, F, G and I. Integrated analysis of glycosidic linkage data from methylation analysis, anomeric configurations, and literature reports suggested the following structures: →4)-α-D-Glcp-(1→ (A) [40], α-L-Araf-(1→ (B) [41], →3,6)-β-D-Galp-(1→ (C) [42], →4)-α-D-GalpA-(1→ (D) [43], →5)-α-L-Araf-(1→ (E) [44], →4)-β-D-Galp-(1→ (F) [45], →6)-β-D-Galp-(1→ (G) [46], →4,6)-α-D-Glcp-(1→ (I) [40]. Residue H could not be fully characterized due to low abundance and weak spectral signals; however, methylation patterns and COSY/HSQC correlations supported its tentative assignment as α-L-Rhap-(1→ [47]. The complete ^1^H/^13^C chemical shift assignments are compiled in Table 2.

Signal assignments of the predominant sugar residues were performed as described below:

Residue A: The anomeric signal at δ 5.33/99.67 (H1/C1) suggested that residue A was likely an α-configured glucopyranosyl residue. Successive COSY cross-peaks enabled the assignment of its ring protons: H2 (δ 3.57) via the δ 5.33/3.57 cross-peak, H3 (δ 3.89) via δ 3.57/3.89, H4 (δ 3.60 via δ 3.89/3.60, H5 (δ 3.77) via δ 3.60/3.77, and H6 (δ3.76) via δ 3.77/3.76. HSQC correlations located C2–C6 at δ 71.71, 73.38, 76.84, 71.18, and 60.45. The downfield shifts observed for C1 and C4 indicated substitution at the O-1 and O-4. On the basis of methylation data and prior reports, residue A was assigned as →4)-α-D-Glcp-(1→.

Residue B: The anomeric signal at δ 5.02/107.52 (H1/C1) indicated that residue B might be an α-configured arabinofuranosyl residue. Sequential COSY cross-peaks enabled full proton assignment: H2 (δ 4.06) via δ5.02/4.06, H3 (δ 3.88) via δ 4.06/3.88, H4 (δ 4.05) via δ 3.88/4.05, and H5 (δ 3.66) via δ 4.05/3.66. Subsequently, HSQC analysis enabled carbon chemical shift assignments: C2–C5 at δ 80.93, 76.75, 83.85, and 61.21. Notably, the downfield shift in C1 indicated substitution at O-1 of the sugar ring. On the basis of methylation data and prior reports, residue B was inferred to be α-L-Araf-(1→.

Residue C: β-Galactopyranose configuration was indicated by the anomeric signal at δ 4.44/102.60 (H1/C1). COSY correlations established the proton assignments: H2 (δ 3.31) via δ 4.44/3.31, H3 (δ 3.65) via δ 3.31/3.65, H4 (δ 4.05) via δ 3.65/4.05, H5 (δ 3.85) via δ 4.05/3.85, and H6 (δ 3.81,3.98) via δ 3.85/3.81, 3.98. Subsequently, HSQC analysis enabled carbon chemical shift assignments: C2 (δ 73.18), C3 (δ 80.23), C4 (δ 68.60), C5 (δ 73.64), C6 (δ 69.44). Diagnostic downfield shifts in C1, C3, and C6 suggested substitution at O-1, O-3, and O-6 positions, respectively. On the basis of methylation data and prior reports, residue C was assigned as →3,6)-β-D-Galp-(1→.

Residue D: The anomeric signal at δ 4.91/98.37 (H1/C1) suggested that residue D might be an α-configured galacturonic acid residue. COSY cross-peaks established sequential proton assignments: H2 (δ 3.54) via δ 4.91/3.54, H3 (δ 3.87) via δ 3.54/3.87, H4 (δ 4.37) via δ3.87/4.37, and H5 (δ 4.70) via δ 4.37/4.70. Subsequently, HSQC analysis enabled carbon chemical shift assignments: C2 (δ 71.86), C3 (δ 68.49), C4 (δ 77.56), C5 (δ 71.20), and C6 (δ 175.04), which is consistent with the presence of a uronic acid residue. The downfield chemical shifts in C1 and C4 suggested substitution at O-1 and O-4, respectively. On the basis of methylation data and prior reports, residue D might be →4)-α-D-GalpA-(1→.

Residue E: α-Linked arabinofuranose was inferred by a characteristic anomeric signal at δ 5.17/109.25 (H1/C1). COSY correlations established the proton assignments: H2 (δ 4.15) via δ 5.17/4.15, H3 (δ 3.94) via δ 4.15/3.94, H4 (δ 3.99) via δ 3.94/3.99 and H5 protons (δ 3.74, 3.82) via δ 3.99/3.74,3.82. Subsequently, HSQC analysis enabled carbon chemical shift assignments: C2–C5 at δ 81.90, 76.75, 84.04, and 66.86. Diagnostic downfield shifts in C1 and C5 suggested substitution at the O-1 and O-5 positions. On the basis of methylation data and prior reports, residue E might be →5)-α-L-Araf-(1→.

Residue F: β-D-Galactopyranose configuration was indicated by anomeric resonances at δ 4.38/103.22 (H1/C1). Sequential COSY correlations established proton assignments: H2 (δ 3.47) via δ 4.38/3.47, H3 (δ 3.70) via δ 3.47/3.70, H4 (δ 4.06) via δ 3.70/4.06, H5 (δ 3.64) via δ 4.06/3.64, and H6 (δ 3.67) via δ 3.64/3.67. HSQC analysis enabled carbon chemical shift assignments: C2 (δ 71.22), C3 (δ 70.13), C4 (δ 76.58), C5 (δ 74.99), C6 (δ 59.34). The diagnostic downfield shifts in C1 and C4 suggested substitution at the O-1 and O-4 positions. On the basis of methylation data and prior reports, residue F was assigned as →4)-β-D-Galp-(1→.

Residue G: β-D-Galactopyranose was indicated by anomeric resonances at δ 4.39/103.44 (H1/C1). COSY correlations established sequential proton assignments: H2 (δ 3.27) via δ 4.39/3.27, H3 (δ 3.51) via δ 3.27/3.51, H4 (δ 3.62) via δ 3.51/3.62, H5 (δ 3.77) via δ 3.62/3.77, and H6 (δ 4.00) via δ 3.77/4.00. HSQC analysis enabled carbon chemical shift assignments: C2 (δ 73.03), C3 (δ 74.28), C4 (δ 72.71), C5 (δ 76.58), C6 (δ 69.71). Diagnostic downfield shifts in C1 and C6 suggested O-1 and O-6 substitution. On the basis of methylation data and prior reports, residue G was assigned as →6)-β-D-Galp-(1→.

Residue H: Based on 1H NMR and COSY spectra, the H6 of residue H was observed at δ 1.19. The cross-peaks at δ 1.19/3.94, 3.94/3.35, and 3.35/3.70 allowed the assignment of H5 (δ 3.94 ppm), H4 (δ 3.35 ppm), and H3 (δ 3.70 ppm). Subsequent HSQC analysis correlated carbon chemical shifts within the sugar ring, revealing C4 at δ 69.45, C5 at δ 68.59 and C6 at δ 16.52. Due to low abundance and weak spectral signals, complete chemical shift assignments could not be achieved. On the basis of methylation data and prior reports, residue H was assigned as →α-L-Rhap-(1→.

Residue I: Residue I was assigned as an α-configured glucopyranosyl unit, supported by the characteristic anomeric signals observed at δ 5.29/99.28 (H1/C1). COSY correlations established sequential proton assignments: H2 (δ 3.51) via δ 5.29/3.51, H3 (δ 3.88) via δ 3.51/3.88, H4 (δ 3.69) via δ 3.88/3.69, H5 (δ 3.80) via δ 3.69/3.80, H6 (δ 3.61) via δ 3.80/3.61. HSQC analysis enabled carbon chemical shift assignments: C2–C6 at δ 71.39, 70.43, 76.14, 71.36, and 66.38. Notably, the diagnostic downfield shifts in C1, C4, and C6 suggested substitution at the O-1, O-4, and O-6 positions. On the basis of methylation data and prior reports, residue I was assigned as →4,6)-α-D-Glcp-(1→.

The linkage patterns of the WDP were deduced from the assigned ^13^C and ^1^H chemical shifts in conjunction with HMBC and NOESY spectral interpretations. In the HMBC spectrum, correlations were observed for A H1/A C4 (δ 5.33/76.84), B H1/E C5 (δ 5.02/66.86), and B C1/E H5 (δ 107.52/3.82). In the NOESY spectrum, cross-peaks were detected for A H1/A H4 (δ 5.33/3.60), B H1/C H3 (δ 5.02/3.65), B H1/E H5 (δ 5.02/3.74 and 5.02/3.82), C H1/C H6 (δ 4.44/3.81 and 4.44/3.98), C H1/F H4 (δ 4.44/4.06), C H1/G H6 (δ 4.44/4.00), D H1/C H6 (δ 4.91/3.81), E H1/C H3 (δ 5.17/3.65), F H1/A H4 (δ 4.38/3.60), and G H1/C H6 (δ 4.39/3.81 and 4.39/3.98). Residue H lacked an anomeric signal and residue I was present at low abundance; therefore, their involvement in the backbone linkages could not be established.

In light of a comprehensive analysis of NMR and methylation data, the WDP was deduced to comprise a backbone formed by the following interlinked residues: →4)-α-D-GalpA-(1→, →3,6)-β-D-Galp-(1→, →6)-β-D-Galp-(1→, →4)-β-D-Galp-(1→ and →4)-α-D-Glcp-(1→ residues. The side chains were formed by terminal α-L-Araf-(1→ residues or arabinan fragments containing α-L-Araf-(1→5)-α-L-Araf-(1→, which were attached to the O-3 position of →3,6)-β-D-Galp-(1→ residues. The inferred WDP structure is illustrated in Figure 4G.

The measured uronic acid content of WDP (21.64% by carbazole-sulfuric acid assay, 12.73% by monosaccharide composition analysis, and 13.1% by methylation analysis), together with the identification of →4)-α-D-GalpA-(1→ residues in its main chain, confirmed that WDP was an acidic polysaccharide. Polysaccharides bearing a β-D-Galp backbone usually have immunomodulatory activity [48]; the polysaccharides isolated from *lactobacillus fermentum* GBJ [49], *Panax notoginseng* residue [50], and processed *Polygonati Rhizoma* with black beans [51] all contain β-D-Galp structures and exhibit good immune-modulating activity. Additionally, polysaccharides with branched structures generally exhibit good immunomodulatory activity [48]. WDP possesses a backbone containing β-D-Galp and features an α-L-Araf-(1→ branched structure; collectively, these findings suggest that WDP has promising immunomodulatory potential, laying a solid foundation for future functional investigations.

### 3.9. Immunomodulatory Activity of WDP

**Figure 5:**
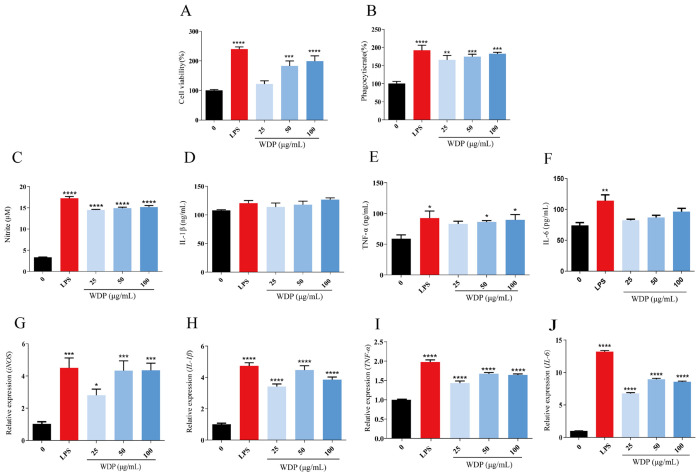
Immunomodulatory activity of WDP on RAW264.7 macrophages: (**A**) cell viability; (**B**) phagocytosis; (**C**–**F**) NO, TNF-α, IL-1β and IL-6 secretion; (**G**–**J**) relative mRNA expression of *iNOS*, *IL-1β*, *TNF-α* and *IL-6*. The data were expressed as the means ± SEM (*n* ≥ 3). * *p* < 0.05, ** *p* < 0.01, *** *p* < 0.001, **** *p* < 0.0001 versus the blank control group.

Macrophages are central mediators of tissue homeostasis and inflammatory immune processes, perform important tissue-specific functions, and protect the organism from infection [52]. As shown in Figure 5A, the WDP (50–100 µg/mL), as well as LPS (1 µg/mL), markedly enhanced the viability of RAW264.7 cells (*p* < 0.001). This marked increase in RAW264.7 cell viability may be related to enhanced metabolic activity and possible proliferation of activated macrophages, which is consistent with previous studies reporting similar increases after LPS or polysaccharide stimulation [53,54,55,56]. Therefore, the results indicated that the WDP was non-cytotoxic within the tested concentration range and may activate RAW264.7 macrophages.

It is known that macrophages can directly phagocytose pathogens, contributing to non-specific immunity and maintaining immune homeostasis in the host. Therefore, phagocytic activity is a critical metric for assessing the immunomodulatory capacity in RAW264.7 cells. As illustrated in Figure 5B, the WDP treatment (25–100 μg/mL) significantly potentiated phagocytosis dose-dependently relative to untreated controls (*p* < 0.01). These data demonstrated that WDP enhanced phagocytic function in RAW264.7 cells, with maximal activity observed at 100 μg/mL. As reported, a polysaccharide (SSP-3a) extracted from the stem and leaves of *Scutellaria baicalensis* [38], as well as neutral oligosaccharides and neutral ginseng polysaccharides from *Panax ginseng* residues [57], also exerted an immunological effect by promoting the proliferation and phagocytic activity of RAW264.7 cells.

NO is a reactive molecule associated with different signaling pathways in organisms [58]. Macrophages are a major source of NO, which is essential for host defense through pro-inflammatory responses [59]. NO can eliminate pathogenic microorganisms and tumor cells [60]. Cytokines, which are broadly secreted proteins, are essential for transmitting cellular signals. By binding to specific receptors on the cell surface, they trigger elaborate downstream signaling cascades that regulate processes including proliferation, differentiation, metabolic activity, and cell survival. A significant proportion of cytokines are produced by immune cells [61]. Extensive research has indicated that polysaccharides derived from plants modulate immune responses by enhancing NO release and modulating the synthesis and secretion of cytokines associated with immune cell polarization [62]. Accordingly, we quantified NO and major pro-inflammatory cytokines in cell culture supernatants following treatment with varying doses of WDP. The results presented in Figure 5C demonstrated that both LPS (1 µg/mL) and varying doses of WDP markedly enhanced NO production relative to the blank control (*p* < 0.0001). This indicated that WDP could potentially regulate immune function through stimulating NO production. In addition, as illustrated in Figure 5D,F, the WDP promoted the secretion of IL-1β and IL-6, and significantly increased TNF-α secretion.

Additionally, to examine the influence of the WDP on the transcriptional levels of *iNOS* and specific cytokines, RT-qPCR was performed. The results depicted in Figure 5G–J indicated that treatment with the WDP (25 to 100 µg/mL) significantly elevated the transcript levels of *iNOS*, *IL-1β*, *TNF-α*, and *IL-6* relative to the blank control. These observations revealed that the WDP mediated the transcriptional upregulation of *iNOS* and key pro-inflammatory cytokines. Notably, the expression of *iNOS* plays a critical role in the host’s defense [63]. Meanwhile, representative pro-inflammatory cytokines like IL-1β, TNF-α, and IL-6 play essential roles in orchestrating both the early inflammatory response and regulating adaptive immunity [64]. Therefore, the above results indicated that the WDP could be developed as a potential immunostimulant in functional foods or as an adjuvant drug. WDP exhibited similar immunomodulatory effects to plant polysaccharides such as *Sambucus adnata* polysaccharides [32], *Gardenia jasminoides* polysaccharide [65] and *Asparagus officinalis* L. polysaccharides [66] that could enhance the immune response by promoting pro-inflammatory cytokine secretion.

**Figure 6:**
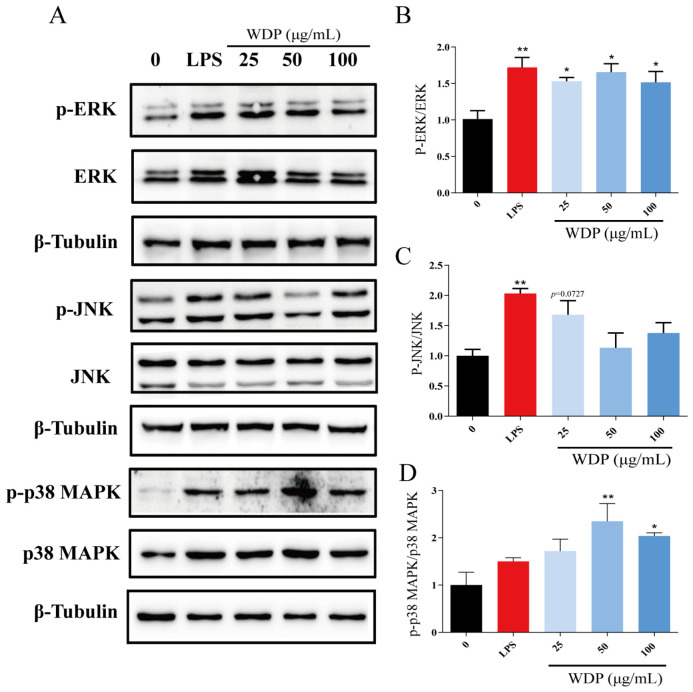
Effect of WDP on MAPK signaling pathway in RAW264.7 macrophages: (**A**) representative Western blot images, (**B**–**D**) quantitative analysis of p-ERK/ERK, p-JNK/JNK, and p-p38/p38. Data were presented as mean ± SEM (*n* = 3). * *p* < 0.05, ** *p* < 0.01 versus the blank control group.

The MAPK pathway is a crucial signaling cascade that mediates cellular processes associated with immune-cell activation, including pro-inflammatory gene expression and cell proliferation [67]. Plant polysaccharides are capable of activating the MAPK signaling cascade through interactions with Toll-like receptors (TLRs). The MAPK family includes ERK, JNK, and p38 MAPK. These proteins are highly conserved serine/threonine kinases across eukaryotes and play central roles in regulating macrophage functions, such as survival, apoptosis, proliferation, differentiation, and immune responses [62]. To determine whether WDP activated RAW264.7 macrophages via the MAPK pathway, WB analysis was conducted to assess ERK, JNK, and p38 MAPK phosphorylation. The results presented in Figure 6 showed that both the LPS- and WDP-treated groups exhibited elevated phosphorylation of ERK, JNK and p38 MAPK compared with the blank control. These results suggested that the WDP had an immunostimulatory function by activating the MAPK signaling pathway. In agreement with this finding, other studies have demonstrated similar mechanisms underlying the immune-modulatory effects of polysaccharides obtained from various plant sources. A polysaccharide (SSP-3) found in *Scutellaria baicalensis* stems and leaves [38], a pectin from *Cucurbita moschata Duch* source [68] and two polysaccharides (Vp2a-II and Vp3) derived from *Apocynum venetum* L. flowers [69] all exerted immune-modulatory effects by activating the MAPK signaling pathway. This study provided further substantiation that polysaccharides derived from plants can activate macrophages via the modulation of the MAPK pathway, thereby exerting immunomodulatory effects.

## 4. Conclusions

In this study, we purified and characterized a novel acidic polysaccharide derived from walnut dregs and named it WDP. Its carbohydrate content reached 90.9%, with uronic acids accounting for 21.64%. The Mw was measured as 351.94 kDa. The WDP consisted primarily of rhamnose, arabinose, galactose, glucose, xylose, mannose, galacturonic acid, and glucuronic acid. Methylation and NMR analyses suggested that the →4)-α-D-GalpA-(1→, →3,6)-β-D-Galp-(1→, →6)-β-D-Galp-(1→, →4)-β-D-Galp-(1→ and →4)-α-D-Glcp-(1→ residues were interconnected to form the backbone of WDP, and its side chains were formed by terminal α-L-Araf-(1→ residues or α-L-Araf-(1→5)-α-L-Araf-(1→ fragments attached to the O-3 position of →3,6)-β-D-Galp-(1→ residues. Functionally, the WDP was found to increase the cell viability and phagocytic activity of RAW264.7 macrophages. In addition, the WDP elevated NO and key cytokine production in RAW264.7 macrophages, thereby triggering an immune response. Meanwhile, the WDP enhanced immunomodulatory activity by activating the MAPK pathway. In summary, WDP holds potential as a novel immunomodulatory agent. The results of this research lay the scientific groundwork for developing immunomodulatory functional foods that utilize walnut dregs as a raw material. In future research, animal experiments can be conducted to further clarify the in vivo immunomodulatory activity, safety, and dose–response relationships of WDP, and the underlying molecular mechanisms should be explored in depth to provide a more comprehensive explanation of WDP’s bioactivity. In addition, the stability of WDP under gastrointestinal conditions and in food systems, as well as its bioavailability, processing suitability, and actual efficacy, should be evaluated to assess its potential as an orally administered functional food ingredient. Meanwhile, the development of WDP-enriched functional food products will facilitate the practical translation of these findings and promote the high-value utilization of walnut processing by-products.

## Figures and Tables

**Table 1:** The methylation result of WDP.

Linkage Type	Partially Methylated Alditol Acetates (PMAAs)	RT	Mass Fragment (*m*/*z*)	Relative Molar Ratio (%)
t-Rha(p)	1,5-di-O-acetyl-6-deoxy-2,3,4-tri-O-methyl mannitol	6.141	72, 89, 102, 115, 118, 131, 162, 175	2.77
t-Ara(f)	1,4-di-O-acetyl-2,3,5-tri-O-methyl arabinitol	6.449	71, 87, 102, 118, 129, 145, 161	15.32
t-Xyl(p)	1,5-di-O-acetyl-2,3,4-tri-O-methyl xylitol	7.858	88, 101, 102, 118, 119, 161, 162	2.19
2-Rha(p)	1,2,5-tri-O-acetyl-6-deoxy-3,4-di-O-methyl mannitol	9.391	89, 100, 115, 130, 131, 175, 190	1.65
t-Man(p)	1,5-di-O-acetyl-2,3,4,6-tetra-O-methyl mannitol	10.596	87, 102, 118, 129, 145, 161, 162, 205	2.88
5-Ara(f)	1,4,5-tri-O-acetyl-2,3-di-O-methyl arabinitol	11.3	87, 102, 118, 129, 162, 189	11.70
3-Gal(p)	1,3,5-tri-O-acetyl-2,4,6-tri-O-methyl galactitol	13.6	87, 101, 118, 129, 161, 202, 234	4.22
4-Gal(p)-UA	1,4,5-tri-O-acetyl-2,3,6-tri-O-methyl galactitol	14.503	87, 99, 102, 115, 118, 131, 162, 175, 235	12.30
4-Gal(p)	1,4,5-tri-O-acetyl-2,3,6-tri-O-methyl galactitol	14.51	87, 102, 113, 118, 129, 162, 233	6.19
4-Glc(p)	1,4,5-tri-O-acetyl-2,3,6-tri-O-methyl glucitol	14.818	87, 102, 113, 118, 129, 162, 233	19.68
2,4-Xyl(p)	1,2,4,5-tetra-O-acetyl-3-O-methyl xylitol	15.085	87, 88, 129, 130, 145, 146, 189, 190	0.90
6-Gal(p)	1,5,6-tri-O-acetyl-2,3,4-tri-O-methyl galactitol	16.262	87, 99, 102, 118, 129, 162, 189, 233	3.13
3,4-Glc(p)-UA	1,3,4,5-tetra-O-acetyl-2,6-di-O-methyl glucitol	16.528	87, 118, 131, 143, 185, 205, 307	0.80
4,6-Glc(p)	1,4,5,6-tetra-O-acetyl-2,3-di-O-methyl glucitol	19.122	85, 102, 118, 127, 159, 162, 201, 261	2.22
3,6-Gal(p)	1,3,5,6-tetra-O-acetyl-2,4-di-O-methyl galactitol	19.738	87, 101, 118, 129, 160, 189, 234	14.05

**Table 2:** Chemical shifts of 1H and 13C of WDP.

Code	Glycosyl Residues	Chemical Shifts (ppm)					
		H1/C1	H2/C2	H3/C3	H4/C4	H5/C5	H6a,b/C6
A	→4)-α-D-Glcp-(1→	5.33	3.57	3.89	3.6	3.77	3.76
		99.67	71.71	73.38	76.84	71.18	60.45
B	α-L-Araf-(1→	5.02	4.06	3.88	4.05	3.66	/
		107.52	80.93	76.75	83.85	61.21	/
C	→3,6)-β-D-Galp-(1→	4.44	3.31	3.65	4.05	3.85	3.81, 3.98
		102.6	73.18	80.23	68.6	73.64	69.44
D	→4)-α-D-GalpA-(1→	4.91	3.54	3.87	4.37	4.7	/
		98.37	71.86	68.49	77.56	71.2	175.04
E	→5)-α-L-Araf-(1→	5.17	4.15	3.94	3.99	3.74, 3.82	/
		109.25	81.9	76.75	84.04	66.86	/
F	→4)-β-D-Galp-(1→	4.38	3.47	3.7	4.06	3.64	3.67
		103.22	71.22	70.13	76.58	74.99	59.34
G	→6)-β-D-Galp-(1→	4.39	3.27	3.51	3.62	3.77	4
		103.44	73.03	74.28	72.71	76.58	69.71
H	α-L-Rhap-(1→	n.d	n.d	3.7	3.35	3.94	1.19
		n.d	n.d	n.d	69.45	68.59	16.52
I	→4,6)-α-D-Glcp-(1→	5.29	3.51	3.88	3.69	3.8	3.61
		99.28	71.39	70.43	76.14	71.36	66.38

Note: “n.d”indicates not detected.

## Data Availability

The original contributions presented in this study are included in the article/Supplementary Materials. Further inquiries can be directed to the corresponding authors.

## Supplementary Materials

The following supporting information can be downloaded at https://www.mdpi.com/article/10.3390/foods15132252/s1. Table S1: Primer Sequences; Figure S1: Mass spectra of partially methylate alditol acetates derived from WDP. [19,21,22,23,24].
