# Supplementary material for: A Novel Polysaccharide from Walnut Dregs: Structural Features and Immunomodulatory Effects via Activation of MAPK Signaling Pathway
Source: Foods. 2026 Jun 23;15(13):2252. doi: 10.3390/foods15132252 (PMC13361250; doi:10.3390/foods15132252)
Supplement: Supplementary file 1 [file foods-15-02252-s001.zip › foods-4321086-supplementary.pdf]

Supplementary Material

2. Materials and methods

2.4. Structural characterization of WDP

2.4.1 Homogeneity and molecular weight

Homogeneity and Mw were measured according to the published methods [21,22]. The WDP was dissolved in 0.1 M $NaNO_3$ containing 0.02% NaN_3 at a concentration of 1 mg/mL and filtered through a filter of 0.45 μm pore size. The homogeneity and molecular weight of WDP were measured by HPSEC using an Alliance e2695 LC instrument（Waters，Corporation, Milford, MA, USA）equipped with a TSKgel GMPWxL (300×7.8mm) combined with Waters 2414 differential refractive index detector (Waters，Corporation, Milford, MA, USA). Chromatographic conditions: mobile phase, 0.1 M $NaNO_3$ and 0.02 % NaN_3; flow rate, 0.5 mL/min; column temperature, 45°C; injection volume, 100 μL. Standard curves generated by the elution time of the Pullulan standards against their concentrations and used to calibrate the M_W of the WDP.

2.4.2 UV-visible spectroscopy

The aqueous solution of WDP (5 mg/mL) was analyzed using a Multiskan GO microplate reader (Thermo Fisher Scientific, Madison, WI, USA) to obtain its UV-vis absorption spectrum across the 200-500 nm range. For reference measurements, stilled water served as the blank control under identical experimental conditions.

2.4.3 Monosaccharide composition analysis

The monosaccharide constituents of WDP were analyzed by the HPAEC-PAD method [23]. About 5 mg of the WDP was hydrolyzed using 2 M trifluoroacetic acid at 121 ^{o}C for 2 hours. The hydrolysate was then dried under a nitrogen stream, followed by 2 times of methanol washes, with drying between each step. The remaining residue was redissolved in deionized water for analysis. For characterization, high-performance anion-exchange chromatography (HPAEC) was employed, utilizing a CarboPac PA-20 column (3 × 150 mm; Dionex, Sunnyvale, CA, USA) coupled with a pulsed amperometric detector (PAD) on a Dionex ICS 5000+ system (Dionex, Sunnyvale, CA, USA). The injection volume was 5 μL. The mobile phases were: H_2O, 0.1 M NaOH, and a mixture of 0.1 M NaOH and 0.2 M NaAc. The flow rate was 0.5 mL/min, and the column temperature was maintained at 30 ^{o}C.

2.4.4. FT-IR spectroscopy analysis

Fourier transform infrared (FT-IR) spectrum of WDP was determined using a spectrometer (Nicolet iZ-10, Thermo Nicolet, Madison, WI, USA) [19]. Briefly, The WDP was homogenized with potassium bromide powder and compressed into a translucent disk (1 mm thick), then analyzed by FT-IR spectroscopy in the mid-infrared region from 4000 to 400 cm^{-1}.

2.4.5. Scanning electron microscope analysis

The microscopic architecture of WDP was examined with a scanning electron microscope(SEM) (Zeiss Merlin Compact, Carl Zeiss, Jena, Germany). After a delicate gold plating, the WDP was affixed to the substrate, and imaged at an accelerating voltage of 1.0 kV. Images were acquired at various magnifications (200x, 500x, and 10000x) within a vacuum-sealed environment.

2.4.6. Methylation analysis

Methylation analysis of WDP followed the procedures outlined in the reference [24]. Briefly, the samples were reduced with $NaBH_4$ and $NaBD_4$, followed by dialysis and lyophilization to obtain the reduced products. These reduced samples were then methylated in a NaOH/DMSO suspension using CH_3I. After thorough methylation, the permethylated derivatives were hydrolyzed with 2 M TFA at 121 °C for 1.5 h. The hydrolysates were subsequently reduced with $NaBD_4$ and acetylated with acetic anhydride at 100 °C for 2.5 h. The resulting acetylated derivatives were dissolved in chloroform and subjected to GC–MS analysis using an Agilent 6890A-5975C system (Agilent Technologies, Santa Clara, CA, USA) equipped with an Agilent BPX70 capillary column (30 m × 0.25 mm × 0.25 μm; SGE Analytical Science, Ringwood, VIC, Australia). High-purity helium was employed as the carrier gas at a split ratio of 10:1, with an injection volume of 1 μL. The temperature program began with an initial hold at 140 °C for 2.0 min, followed by an increase to 230 °C at a rate of 3 °C/min, and a final hold at 230 °C for 3 min. Mass spectrometry detection was carried out in SCAN mode, covering a mass range from m/z 50 to m/z 350.

2.4.7. NMR spectroscopy analysis

The WDP was dissolved in D_2O at a final concentration of 40 mg/mL and subsequently transferred to an NMR tube. Systematic NMR analysis of WDP, including 1D (^1H-NMR, ^{13}C-NMR) and 2D (COSY, NOESY, HMBC, HSQC) spectra, was performed using a Bruker AVANCE NEO 500 M spectrometer (Bruker BioSpin GmbH,, Rheinstetten, Germany) to obtain complete structural information. All NMR spectra were acquired with the scanning temperature controlled at 25℃.

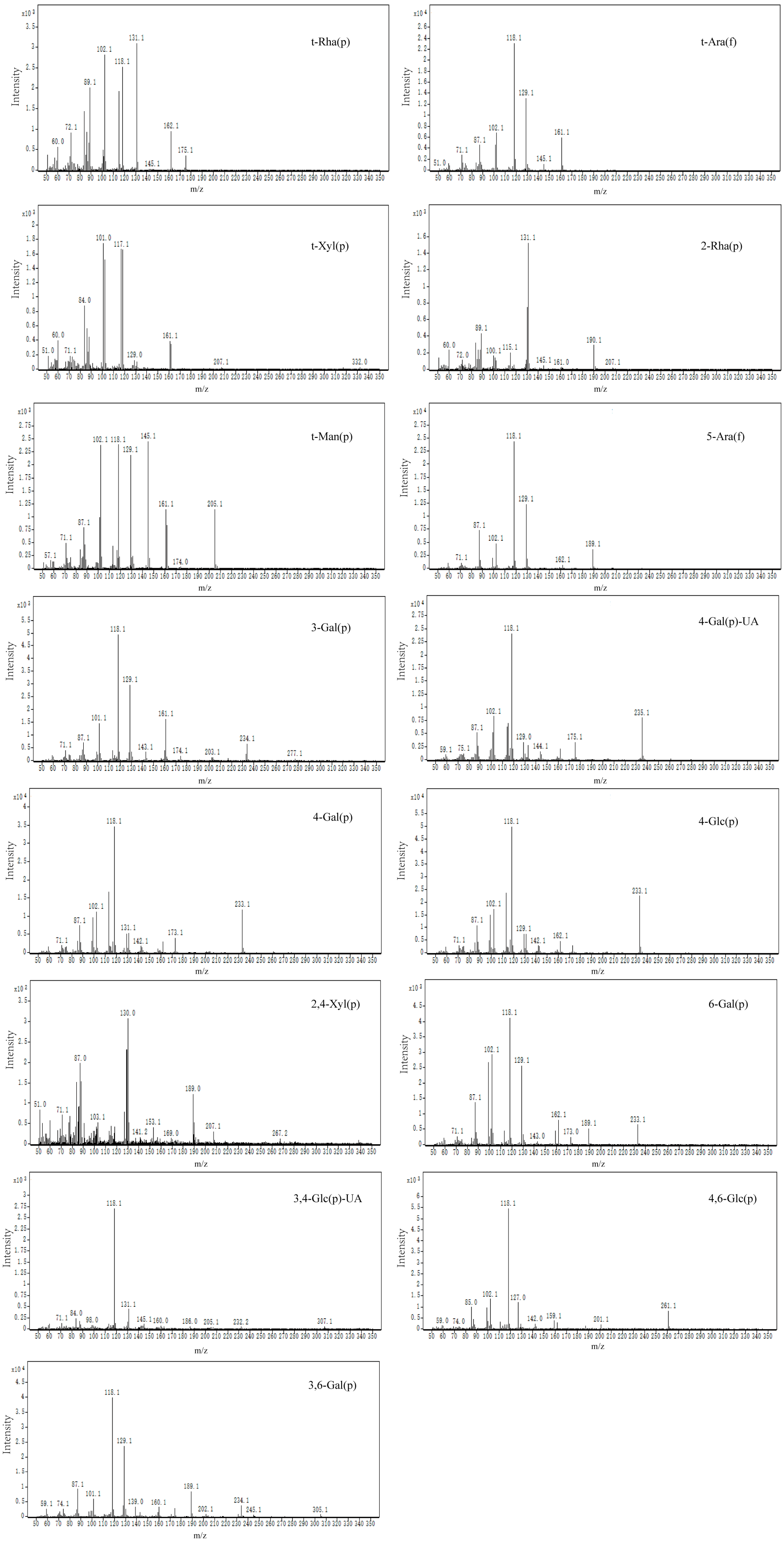

Figure S1. Mass spectra of partially methylated alditol acetates derived from WDP

Table S1 Primer Sequences

Primer	Forward
β-actin/F	ACGGCCAGGTCATCACTATTG
β-actin/R	TGGAAAAGAGCCTCAGGGC
TNF-α/F	ACACCGTCAGCCGATTTGCTA
TNF-α/R	CCAAAGTAGACCTGCCCGGAC
IL-6/F	AGTTGCCTTCTTGGGACTGA
IL-6/R	TTCTGCAAGTGCATCATCGT
IL-1β/F	GATGAAGGGCTGCTTCCAAC
IL-1β/R	GCTTCTCCACAGCCACAATG
iNOS/F	ACATTCAGATCCCGAAACGC
iNOS/R	GACAATCCACAACTCGCTCC
